# NS1 and PA-X of H1N1/09 influenza virus act in a concerted manner to manipulate the innate immune response of porcine respiratory epithelial cells

**DOI:** 10.3389/fcimb.2023.1222805

**Published:** 2023-07-26

**Authors:** Robin Avanthay, Obdulio Garcia-Nicolas, Gert Zimmer, Artur Summerfield

**Affiliations:** ^1^ Institute of Virology and Immunology, Mittelhäusern, Switzerland; ^2^ Department of Infectious Diseases and Pathobiology, Vetsuisse Faculty, University of Bern, Bern, Switzerland; ^3^ Graduate School for Cellular and Biomedical Sciences, University of Bern, Bern, Switzerland

**Keywords:** influenza A virus, NS1 protein, PA-X protein, host shut-off, cytokine, innate immune response, transcriptomic profiling, virus attenuation

## Abstract

Live-attenuated influenza A viruses (LAIV) may be superior to inactivated or subunit vaccines since they can be administered via mucosal routes to induce local immunity in the respiratory tract. In addition, LAIV are expected to trigger stronger T-cell responses that may protect against a broader range of antigen-drifted viruses. However, the development of LAIV is challenging since a proper balance between immunogenicity and safety has to be reached. In this study, we took advantage of reverse genetics to generate three LAIV based on the pandemic H1N1 2009 (pH1N1/09) virus strain: ΔPA-X, which is defective in the synthesis of the accessory PA-X protein, NS1(1-126) lacking 93 amino acids at the C-terminus of the NS1 protein, and a combination of both. Characterization of these recombinant viruses using a novel porcine bronchiolar epithelial cell line (T3) revealed that the ΔPA-X mutant replicated similar to wild type (WT) virus. However, in contrast to the parental virus the ΔPA-X mutant allowed transcription of genes involved in cell cycle progression and limits apoptosis. The NS1(1-126) mutant also replicated comparable to WT virus, but triggered the release of type I and III IFN and several chemokines and cytokines. Surprisingly, only the NS1(1-126)/ΔPA-X double mutant was significantly attenuated on T3 cells, and this was associated with enhanced transcription of genes of the innate immune system and complete absence of apoptosis induction. In conclusion, these findings indicate that NS1 and PA-X act in a concerted manner to manipulate the host cell response, which may help to develop swine LAIV vaccine with a more favorable balance of safety and immunogenicity.

## 1 Introduction

Swine influenza A viruses (SIAV) are frequently circulating in pig farms worldwide leading to respiratory disease and significant economic losses. Pigs are also susceptible to both human and avian influenza viruses and have been regarded as a “mixing vessel” where reassortment of gene segments may occur (Scholtissek, 1995). These reassortant viruses shall have new antigenic properties and – following transmission to humans – have the potential to cause a pandemic in the naive human population. In fact, the 2009 H1N1 pandemic virus (pH1N1/09), a triple reassortant virus with gene segments derived from human, porcine and avian IAV (Hajjar and McIntosh, 2010; Mena et al., 2016), is an example for such an event. It is believed that vaccination of pigs may not only reduce the burden of disease in the animals but may also lower the risk of emergence of potentially pandemic viruses.

Inactivated whole influenza virus vaccines (WIV) are already in use for the immunization of SIAV (Gasparini et al., 2011). Although these vaccines can reduce the morbidity of influenza disease in pigs, they also have limitations. Inactivated vaccines do not efficiently stimulate the cellular arm of the immune system and mostly rely on the induction of antibodies that are specifically directed to the variable envelope antigens HA and NA. Consequently, WIV-induced immune responses are specifically directed against the antigens of the virus strain from which the vaccine is derived from, but may not protect against antigen-drifted IAV strains. Moreover, WIV are usually administered via the parenteral route, and trigger the synthesis of serum antibodies which are only partially secreted into the mucosal tissues of the respiratory tract. Therefore, WIV fail to induce sterilizing immunity and do not completely abrogate shedding of influenza viruses from the upper respiratory tract (Heinen et al., 2001; Vincent et al., 2008; Vincent et al., 2010). In contrast, vaccines based on live-attenuated influenza viruses (LAIV) are administered via the intranasal route, which triggers mucosal immune responses that are able to neutralize virus directly at the entry site (Laurie et al., 2010; Dutta et al., 2016). Moreover, LAIV activate the cellular arm of the immune system and therefore act in a more broadly protective manner (Cheng et al., 2013; Janssens et al., 2022). However, it has always been challenging in the past to find the right balance between sufficient attenuation and the remaining immunogenicity of LAIV.

Influenza A viruses expressing a modified nonstructural protein 1 (NS1) have attracted a lot of interest as LAIVs for veterinary use (Nogales et al., 2022). The NS1 protein is a multifunctional protein that has been shown to antagonize type I interferon (IFN-I) production by inhibiting activation of the RIG-I/TRIM25 complex (Kochs et al., 2007), blocking activation of the NFκB transcription factor (Geiss et al., 2002), and by interacting with the host factor CPSF30 (Nogales et al., 2018; Ji et al., 2021). The binding to the cellular 30 kDa subunit of CPSF leads to inhibition of 3’ end formation of cellular pre-mRNAs leading to inhibition and global deregulation of host transcription (host shut-off) (Nemeroff et al., 1998; Nacken et al., 2021). Interestingly, some IAV strains such as pH1N1/09 bind less efficiently to CPSF30 (Clark et al., 2017) and show reduced host shut-off activity, which might explain the relatively mild clinical disease caused by pH1N1/09 (Swedish, 2011). NS1 is also known to block the antiviral effects of IFN-induced antiviral proteins, such as dsRNA-dependent protein kinase R (PKR) and 2’5’-oligoadenylate synthetize (OAS)/RNase L (Min and Krug, 2006; Min et al., 2007; Nogales et al., 2018).

Three functionally distinct domains have been deciphered in the NS1 protein: The N-terminal RNA binding domain (RBD, amino acid 1-73), the effector domain (amino acid 74-207), and the “disordered tail” (amino acid 208-230) (Kim et al., 2021). Analysis of A/Swine/Texas/4199-2/98 (H3N2) (TX/98) with C-terminal truncated NS1 protein (1-73, 1-99, or 1-126) revealed a decreased ability of the recombinant viruses to block IFN-I and IFN-III synthesis, consequently showing an attenuated phenotype in pigs (Solórzano et al., 2005). Subsequent studies demonstrated that intranasal administration of TX/98-NS1(1-126) triggered a mucosal immune response that lead to complete protection against homologous challenge and nearly complete protection against an antigenic H3N2 variant (Richt et al., 2006; Vincent et al., 2007). In 2017, the Tx/98-NS1(1-126) LAIV was licensed in the US for the prevention of influenza in pigs from one day of age and was shown to efficiently reduce nasal shedding after challenge with heterologous IAV strains (Genzow et al., 2018; Kaiser et al., 2019). However, reassortant strains containing LAIV genes in combination with genes from endemic field strains were detected, indicating a substantial degree of LAIV replication and shedding (Mancera Gracia et al., 2020; Sharma et al., 2020).

Apart from NS1, the PA-X protein has also been found to mediate host shut-off activity (Firth et al., 2012). PA-X is translated from an overlapping protein-coding region in IAV segment 3. It is expressed at low levels due to the low efficiency of the ribosomal frameshift (Muramoto et al., 2013). PA-X is composed of the 191 N-terminal amino acids of the PA protein but has a distinct C-terminal sequence of 61 amino acids (Muramoto et al., 2013; Chaimayo et al., 2018). Some IAV, including pH1N1/09, have a truncated form of PA-X lacking 20 amino acids at the C-terminus (Lee et al., 2017). PA-X specifically degrades host mRNA, in particular the mRNA of cellular RNA polymerase II, without affecting viral mRNA (Gaucherand et al., 2019). Recent findings suggest that NS1 and PA-X act in a concerted manner in order to manipulate the host innate immune response (Nogales et al., 2017; Nogales et al., 2018; Gaucherand et al., 2019; Dunagan et al., 2021).

In view of the safety issues associated with swine LAIV vaccines, the present study aimed to identify a strategy to further attenuate SIAV replication but retaining its capacity to stimulate the immune system. To this end, we generated different recombinant porcine originated pH1N1/09 viruses which include a truncated NS1 protein, a knock-out of the PA-X protein, or a combination of both. Using the recently established porcine bronchiolar epithelial porAEC (“T3”) cell line (López-Gálvez et al., 2021; Fleurot et al., 2022), we compared the recombinant viruses with respect to attenuated replication and the induction of chemokines and cytokines such as IFN-I and IFN-III. Our data support the idea that both NS1 and PA-X contribute to the viral host shut-off activity and may explain why the previously licensed NS1-truncated LAIV were not sufficiently attenuated. We propose that the NS1(1-126)/ΔPA-X mutant virus might be a promising swine LAIV vaccine candidate which deserves further evaluation *in vivo*.

## 2 Materials and methods

### 2.1 Cells

The T3 porcine bronchiolar epithelial cell line was generated by InSCREENeX GmbH (Braunschweig, Germany) and kindly provided by Sasha Trapp (Institut national de la recherche agronomique, INRA, France). The cells were maintained at 37°C and 5% CO_2_ in porAEC medium with supplement (InSCREENeX, INS-ME-1024) in collagen-coated (InSCREENeX, INS-SU-1017) flasks. Madin-Darby canine kidney type II cells (MDCK-II) were kindly provided by Georg Herrler (University of Veterinary Medicine, Hannover, Germany) and maintained at 37°C and 5% CO_2_ with minimum essential medium (MEM, Gibco) supplemented with 5% of fetal bovine serum (FBS). Human embryonic kidney (HEK) 293T cells (ATCC) were maintained at 37°C and 5% CO_2_ with Dulbecco’s Modified Eagle Medium (DMEM, Gibco) supplemented with 10% of fetal bovine serum (FBS). Human type II pneumocyte lung tumor cells (A549) (Sigma-Aldrich) were maintained at 37°C and 5% CO_2_ in Ham’s F12 medium (Gibco) supplemented with 5% of fetal bovine serum (FBS).

### 2.2 Generation of recombinant influenza viruses

The pHW2000 plasmids encoding the 8 segments of A/Hamburg/4/2009 (H1N1) were kindly provided by Martin Schwemmle (Institute of Virology, University of Freiburg, Germany). The pHW2000-NS plasmid encoding the genomic segment 8 was modified by introducing 4 stop codons into the NS1 open reading frame as previously described (Solórzano et al., 2005), resulting in the C-terminally truncated NS1(1-126) protein. The pHW2000-ΔPA-X plasmid was generated by modifying the nucleotide sequence 592-UCCUUUCGU-600 in the PA gene to 592-UCGUUCAGA-600, following a published strategy (Nogales et al., 2017; Chaimayo et al., 2018). For generation of recombinant virus, a co-culture of MDCK-II and HEK 293T cells was transfected with Lipofectamine 2000 (Life Technologies) and a mixture of the pHW2000 plasmids encoding the 8 viral genomic segments (2 µg of each plasmid). Following incubation of the cells for 24 hours, the medium was replaced by Opti-MEM medium (Life Technologies) containing 1% penicillin/streptomycin and 1 µg/ml of N-tosyl-L-phenylalanine chloromethyl ketone-treated trypsin (TPCK-trypsin, Merck KGgA). The following day, half of the medium was replaced by DMEM containing 0.2% (w/v) of bovine albumin serum (BSA) and 1 µg/ml of TPCK-trypsin. Following an incubation of the cells for 24 hours, the cell culture supernatant was collected, and cell debris removed by low-speed centrifugation. For production of stocks, the viruses were passaged twice on MDCK-II cells in the presence of TPCK-trypsin. Two days post infection (p.i.), the cell culture supernatant was collected, FBS was added (5% final concentration), and cell debris removed by centrifugation. Viruses were aliquoted and stored at -70°C.

The recombinant viruses were titrated on MDCK-II cells grown in 96-well cell culture plates. The viruses were serially diluted (10-fold dilution steps) and 40 µl of each dilution was added to the wells in quadruplicates. Following an incubation for 90 minutes at 37°C, 160 µl of MEM medium containing 1% (w/v) of methylcellulose were added to each well and the cells incubated for 24 hours at 37°C in a 5% CO_2_ atmosphere. The cells were fixed with 3.7% formalin in PBS (w/v), permeabilized with PBS containing 0.25% of Triton X-100 (v/v), and infected cells detected by indirect immunofluorescence using a monoclonal antibody directed to the influenza virus nucleoprotein (Ocaña-Macchi et al., 2009). Tissue culture infectious dose 50% (TCID_50_)/ml was calculated according to the Reed-Muench formula (Lei et al., 2020).

### 2.3 Virus replication kinetics

To evaluate the replication kinetics of the viruses, MDCK-II, T3 and A549 cells were grown in 6-well cell culture plates and inoculated (3 replicates) with virus for 90 minutes using a multiplicity of infection (MOI) of 0.0001 TCID_50_/cell. Thereafter, the inoculum was removed, the cells washed once, and maintained in 2.5 ml/well of FBS-deficient MEM medium supplemented with 1% (v/v) penicillin/streptomycin, 1 µg/ml of TPCK-trypsin, either in the presence or absence of 1 µM of ruxolitinib (Selleckchem). At the indicated time points p.i., 250 µl of cell culture supernatant were collected and titrated on MDCK-II cells as described in section 2.2.

### 2.4 Plaque forming assay

To evaluate the plaques characteristics induced by the viruses, MDCK-II and T3 cells were grown in 24-well cell culture plates and inoculated (3 replicates) with virus for 90 minutes using serial diluted virus from 10^5^ to 10^2^ TCID_50_/ml. Thereafter, the inoculum was removed, the cells washed once, and maintained for 72 hours in 1ml/well of FBS-deficient MEM medium containing 1.2% Avicel (RC-581NF, FMC Biopolymer) supplemented with 1 µg/ml of TPCK-trypsin. Plaques were stained as described in section 2.2. Immunofluorescence images were acquired using a Lionheart FX automated microscope (BioTek) and area was determined using Image J software (Version 1.53t, NIH).

### 2.5 RNA extraction and RT-qPCR

Cells grown in 24-well cell culture plates were inoculated with virus using an MOI of 1 TCID_50_/cell. Following an incubation for 90 minutes at 37°C, the inoculum was removed, the cells washed three times with PBS, and fresh MEM medium added. The cells were lysed using RAI lysis buffer (Macherey-Nagel). RNA extraction from cell lysates was performed using the NucleoMag Vet kit (Macherey-Nagel) according to the manufacturer’s protocol. Reverse transcription from RNA to cDNA and real time quantitative PCR (qPCR) was performed with the AgPath-ID™ One-Step RT-PCR kit (Life Technologies) using the 7500 Real-Time PCR System (ThermoFisher). Data were acquired and analyzed using the 7500 Fast System SDS software v1.4 (Fisher Scientific) according to the double delta Ct method (2^-ΔΔCT^) (Livak and Schmittgen, 2001). The endogenous reference gene 18S was used for normalization. Oligonucleotide and probes used in this study are listed in **Supplementary Table 1**.

### 2.6 Western blot analysis

MDCK-II cells were grown in 6-well cell culture plates and infected at an MOI of 3 TCDI_50_/cell and cultured for 20 hours at 37°C. Infected cells were lysed using NP40 lysis buffer (ThermoFisher, cat. No. J60766.AP), containing protease inhibitor cocktail (cOmplete, Merck cat. N° 04693132001). The cell lysate proteins were separated by SDS-PAGE containing a gradient from 4-12% acrylamide (Gene script, cat. No. M00653), in a reducing condition, and then transferred to nitrocellulose membranes. The blots were incubated overnight at 4°C with blocking buffer (LI-COR Biosciences) and subsequently 60 minutes incubation with mouse anti-influenza NS1 N-terminus (1:200, Santa Cruz Biotechnology, cat. no. sc-130568). The blots were washed 4 times with PBS containing 0.1% (v/v) Tween 20 and thereafter incubated in the dark for 60 minutes with goat anti-mouse IgG IRDye hb800CW (LI-COR, cat. no. 926-32210). Following several wash steps with PBS containing 0.1% (v/v) Tween 20, the blots were scanned with the Odyssey Infrared Imaging system (LI-COR Biosciences) and the scans analyzed by Image Studio version 5.2 software (LI-COR Biosciences).

### 2.7 RNA sequencing and analysis

T3 cells grown in 24-well plates were inoculated for 90 minutes at 37°C with the recombinant influenza viruses using an MOI of 1 TCID_50_/cell. Thereafter, the inoculum was removed and the cells were washed twice with warm PBS, fresh medium was added, then cells were returned to the CO_2_ incubator at 37°C. At 24 or 48 hours p.i., the cells were washed twice and RNA was extracted using TRIzol Reagent (Life Technologies). The RNA quality was assessed by fragment analysis (5200 Fragment Analyzer CE instrument, Agilent) and sequencing was performed using an Illumina® NovaSeq6000 sequencer (Illumina). Data analysis was performed with R software version 4.2.1 (2022-06-23) (R Core Team 2022). Differential gene expression (DGE) analysis of the experimental groups was performed using the Bioconductor software package DESeq2 v1.36.0. Venn diagrams were created using the list of differentially expressed genes uploaded on an online Venn diagram tool (https://bioinformatics.psb.ugent.be). ClusterProfiler v.4.4.4 (Wu et al., 2021) was used to identify gene ontology terms that were significantly modulated. Gene set enrichment analysis (GSEA) (Subramanian et al., 2005) was performed with ClusterProfiler v4.4.4 (Wu et al., 2021) using MSigDb (Liberzon et al., 2015).

### 2.8 Caspase 3/7 enzyme assay and LDH cytotoxicity assay

As described above, T3 cells were seeded in 96-well plates and inoculated for 90 minutes at 37°C with recombinant influenza viruses using an MOI of 1 TCID_50_/cell. The inoculum was removed, the cells washed three times with PBS and then further maintained at 37°C in a 5% CO_2_ atmosphere. At 24, 48, and 72 hours p.i., caspase 3/7 activity was monitored in cell lysates using the Caspase-Glo-3/7 kit (Promega; cat. no. G8090) according to the manufacturer`s instructions. LDH level was monitored in the supernatant using the LDG-Glo-Cytotoxicity assay kit (Promega; cat. no. J2380) according to the manufacturer’s instructions.

### 2.9 Statistics

Data analysis and figures were done using GraphPad Prism 8 Software (GraphPad Software). Multiple comparison one-way and two-way ANOVA test were used to determine statistical significance in terms of gene expression, viral titers, and protein activity. P value lower than 0.05 was considered as statistically significant.

## 3 Results

### 3.1 Virus replication of the NS1(1-126)/ΔPA-X mutant is attenuated in T3 cells

**Figure 1:**
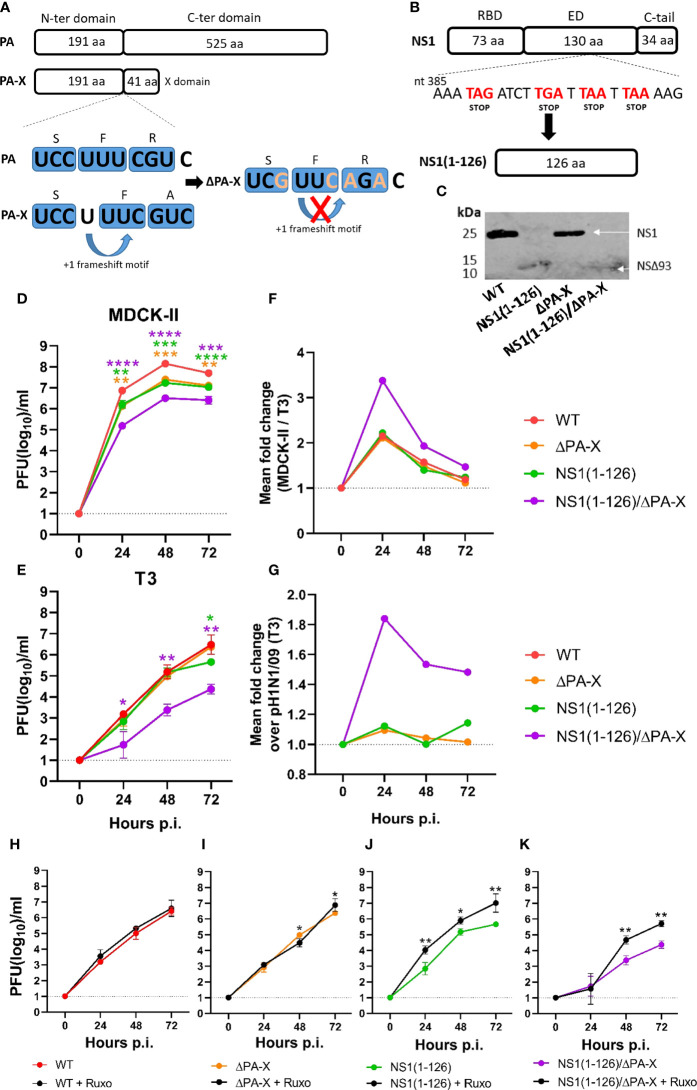
Replication of NS1 and PA-X mutants in MDCK-II and T3 cells. **(A)** Schematic representation of the PA and PA-X proteins and the mutations (orange letters) introduced into the frameshift motif to inhibit PA-X synthesis. **(B)** Schematic representation of NS1 protein and the inclusion of four stop codons (red letters) resulting in the truncated NS1(1-126) protein. **(C)** MDCK-II cells were infected with the indicated viruses using an MOI of 3 TCID_50_/cell and lysed 20 hours p.i. The NS1 and NS1(1-126) proteins were detected by Western blot using a monospecific antibody directed to the NS1 N-terminus. **(D)** MDCK-II and **(E, H–K)** T3 cells were infected with the indicated viruses using an MOI of 0.0001 TCID_50_/cell and maintained at 37°C in the presence or absence of ruxolitinib. At the indicated times, cell culture supernatant was collected and infectious virus titers determined. **(F)** Fold change in MDCK-II over T3 cells. **(G)** Fold change in T3 cells infected with the mutant viruses compared to WT virus. Significant differences were determined comparing WT to NS1(1-126), ΔPA-X, or NS1(1-126)/ΔPA-X **(D–E)** and each virus with ruxolitinib compared to the same virus without ruxolitinib using the two-way ANOVA test **(H–K)**. *p<0.05, **p<0.01, ***p<0.001, ****p<0.0001 indicate significant difference.

Using reverse genetics, we produced three mutant viruses based on the pandemic virus A/Hamburg/4/2009(H1N1) which lacked either the PA-X protein (ΔPA-X), the last 93 amino acids of the NS1 protein [NS1(1-126)], or a combination of NS1(1-126)/ΔPA-X mutations (**Figures 1A, B**). The mutations were confirmed by Sanger sequencing of the targeted genome segments. Western blot analysis of Madin-Darby canine kidney cell type II (MDCK-II) infected cell lysates indicated a molecular weight shift for the NS1(1-126) protein (**Figure 1C**). We speculate that the stability of this truncated protein is highly compromised and subject to faster degradation than full-length NS1 protein which explain the weaker expression compared to WT NS1. Unfortunately, we couldn’t detect the PA-X protein in wild-type virus infected cells, most likely because of low expression levels, high turnover rates (Levene et al., 2021) and no specific available antibody against pH1N1/09 PA-X.

Virus replication kinetics were analyzed using two different cell lines, MDCK-II cells, a commonly used cell line for influenza A virus replication (Zhai et al., 2012), and the more relevant porcine bronchiolar epithelial cell-derived T3 cells (López-Gálvez et al., 2021; Fleurot et al., 2022). In fact, transcriptomic profiling of T3 cells demonstrated their similarity to primary upper respiratory tract epithelial cells (**Supplementary Figure 1**).

Both cell types were infected with the recombinant pH1N1/09 mutants using an MOI of 0.0001 TCID_50_/cell. Virus released into the cell culture supernatant was collected at 0, 24, 48, and 72 hours post infection. (p.i.), and the infectious titers determined. In MDCK-II cells, infectious titers of both NS1(1-126) and ΔPA-X were reduced by one log_10_ compared to WT virus (**Figure 1D**). For the double mutant NS1(1-126)/ΔPA-X, titers dropped by two log_10_ when compared to WT pH1N1/09 (**Figure 1D**). In T3 cells, all recombinant viruses replicated slower and to generally lower titers than in MDCK-II cells (**Figures 1E, F**). The ΔPA-X mutant did not show any signs of attenuation and NS1(1-126) showed a drop of infectious titer only at 72 hours p.i. (**Figures 1E, G**). Similar to what has been observed in MDCK-II cells, the NS1(1-126)/ΔPA-X mutant was the most attenuated virus reaching only 10^4^ pfu/ml at 72 hours p.i. (**Figures 1E, G**). Replication of WT virus and ΔPA-X mutant in T3 cells was not affected by ruxolitinib (**Figures 1H, I**), an inhibitor of the JAK1 and 2 signaling pathway (Verstovsek, 2009). However, replication of NS1(1-126) and NS1(1-126)/ΔPA-X was partially rescued in the presence of the inhibitor, reaching similar titers as WT virus (**Figures 1J, K**). These findings indicate that both NS1 and PA-X protein need to be modified in order to suppress virus replication in T3 cells.

The attenuation of the NS1(1-126) and NS1(1-126)/ΔPA-X mutants was also evident from the smaller size of plaques that were formed and/or lower stained antigen expressed by these viruses in MDCK-II cells (**Supplementary Figures 2A, B**), as well as from replication kinetics performed in A549 cells (**Supplementary Figure 2C**), a human type II pneumocyte-derived tumor cell line, which has been frequently used to study influenza A virus infection in human (Giard et al., 1973; Ujie et al., 2019). Cell infection but no plaques formation in T3 cells highlight the more resistant phenotype of these cells to influenza infection

### 3.2 NS1(1-126) and ΔPA-X mutants show distinct transcriptomic profiles in T3 cells

**Figure 2:**
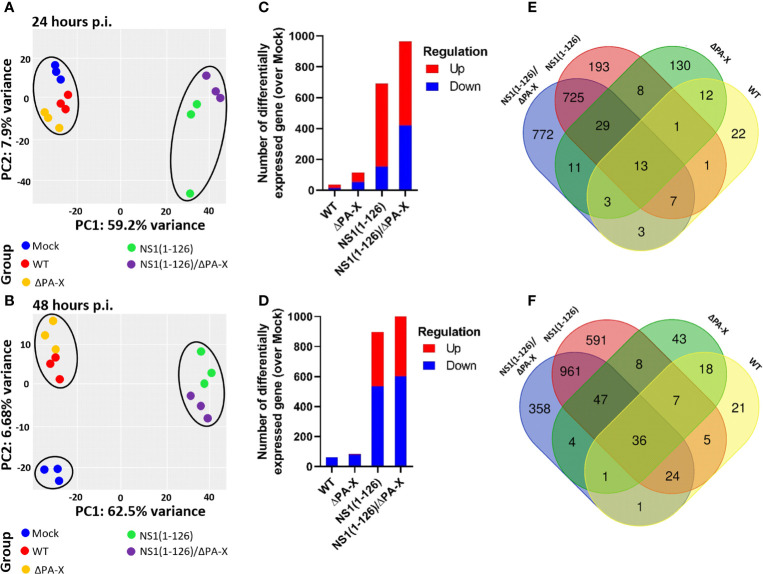
General effects of NS1 and PA-X on T3 cell gene expression. **(A, B)** PC1 and PC2 analysis of T3 cells at 24 hours **(A)** and 48 hours **(B)** p.i. with either WT, ΔPA-X, NS1(1-126) or NS1(1-126)/ΔPA-X virus. **(C, D)** Number of differentially expressed genes (DEGs) in infected cells over Mock infected cells at 24h p.i. **(C)** and 48 hours p.i. **(D)**. **(E, F)** Venn diagrams showing the overlap of DEGs in T3 cells at 24 hours p.i. **(E)** and 48 hours p.i. **(F)** with the indicated viruses.

To study the cellular response, T3 cells were infected with WT and mutant viruses at an MOI of 1 TCID_50_/cell and the transcriptome of the cells was determined at 24 and 48 hours p.i. by RNA sequencing. These time points were chosen since RT-qPCR analysis did not indicate earlier induction of the IFNL3 and MX1 gene transcription (**Supplementary Figure 3A**). At 24 hours p.i., principal component analysis (PCA) showed clustering of WT pH1N1/09 and ΔPA-X mutant virus with mock-infected cells, but clear separation from NS1(1-126) and NS1(1-126)/ΔPA-X with respect to principal component 1 (PC1) (**Figure 2A**). At 48 hours p.i., the pronounced effect by the NS1(1-126) and NS1(1-126)/ΔPA-X mutants on PC1 were maintained, but now the WT and ΔPA-X mutant viruses also clearly separated from mock-treated cells on PC2 (**Figure 2B**).

In addition to PCA, differentially expressed genes (DEGs) were compared in infected over mock-treated cells. While WT virus and ΔPA-X mutant induced only a few DEGs (**Figures 2C, D**), the NS1(1-126) mutant and in particular the NS1(1-126)/ΔPA-X mutant triggered the induction of a high number of DEGs in T3 cells (**Figures 2C, D**). At 48 hours p.i., the number of downregulated genes increased, especially in NS1(1-126) and NS1(1-126)/ΔPA-X infected cells (**Figure 2D**). A heatmap of the 500 most significantly differentially expressed genes showed clustering between mock, WT and ΔPA-X on the one hand and between NS1(1-126) and NS1(1-126)/ΔPA-X on the other hand (**Supplementary Figure 4**). Venn diagrams demonstrated the pivotal role of the NS1(1-126) mutant in triggering transcriptional changes at 24 and 48 hours p.i., and also highlighted the distinct effect of the ΔPA-X mutant on T3 gene expression (**Figures 2E, F**). At 24 hours p.i. in particular, the majority of the ΔPA-X induced DEGs was found to be unique to this mutant (**Figure 2E**). More than 50% of the differentially expressed genes in NS1(1-126)/ΔPA-X infected cells were not shared by cells infected with the NS1(1-126) mutant (**Figure 2E**).

### 3.3 NS1(1-126) and NS1(1-126)/ΔPA-X mutants induce a robust antiviral and inflammatory response in T3 cells

**Figure 3:**
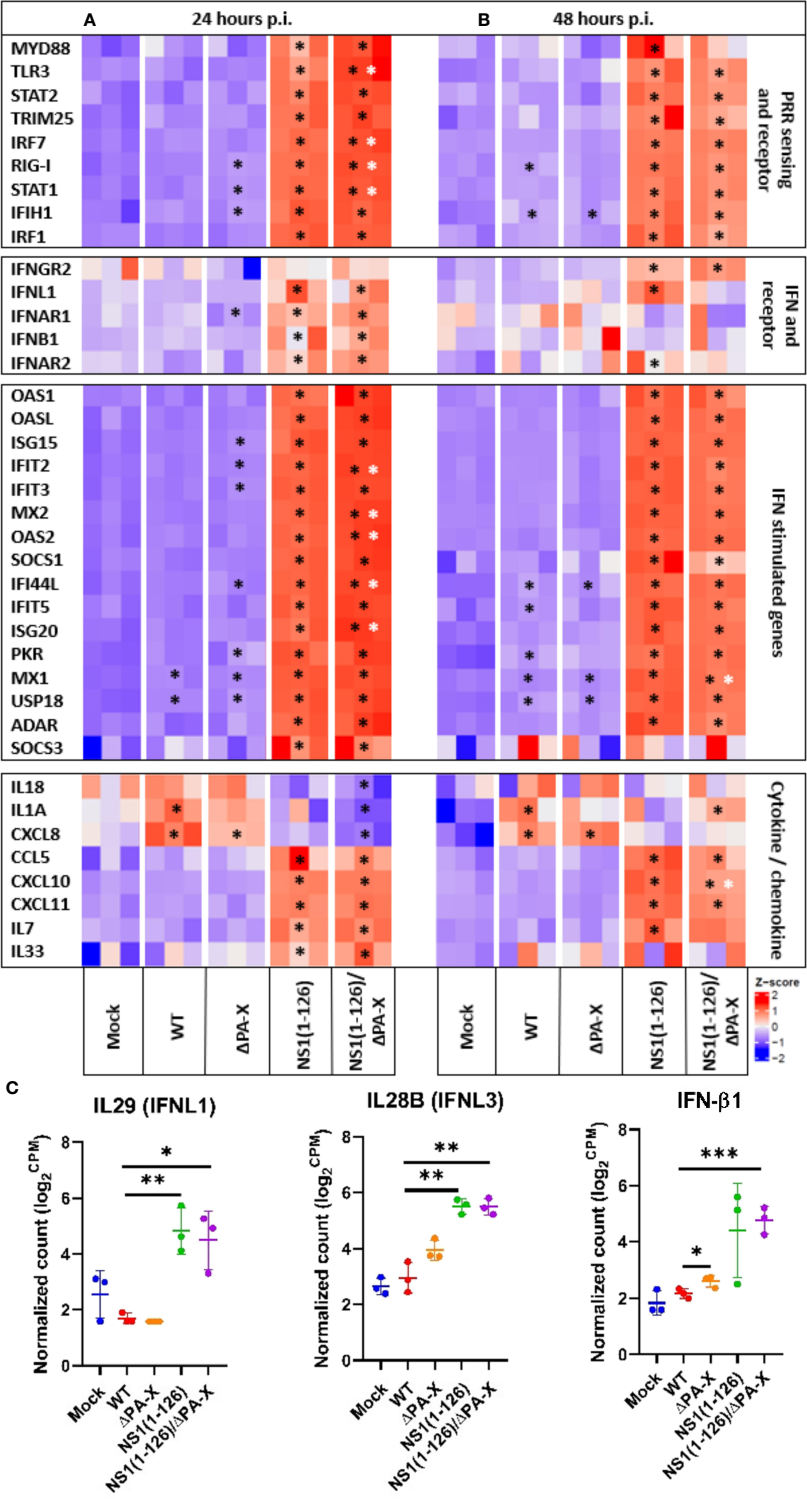
Relative expression levels of genes of the innate immune system in infected T3 cells. Heatmap illustrating the relative expression levels of genes of the innate immune system including PRR sensing and adapter genes, IFN and IFN receptor genes, IFN-stimulated genes and various cytokine and chemokine genes of T3 cells 24 hours **(A)** and 48 hours **(B)** p.i. Genes from infected cells showing significant differences to mock-infected cells are indicated by black stars, while genes of NS1(1-126)/ΔPA-X infected cells showing significant differences compared to NS1(1-126) infected cells are indicated by white stars. The Z score of each heat map is represented in the lower right corner. The adjusted p-value of each comparison is shown in **Supplementary Tables 2**, **3**. **(C)** Normalized count in log_2_ count per million (CPM) obtained from the transcriptomic reads count at 24 hours p.i. *p<0.05, **p<0.01, ***p<0.001, indicate significant difference.

A volcano plot pointed out that most of the transcripts that were induced in T3 cells by the NS1(1-126) and NS1(1-126)/ΔPA-X mutants belong to the IFN responsive gene cluster, while this response appeared to be absent in the WT and ΔPA-X infected T3 cells (**Supplementary Figures 5**, **6**). Analysis of genes involved in pathogen sensing, IFN responses, and inflammation revealed that the transcription of genes involved in RNA sensing was upregulated in T3 cells only if the cells were infected with NS1(1-126) or NS1(1-126)/ΔPA-X, whereas infection with either WT pH1N1/09 or ΔPA-X mutant did not enhance transcription of these genes (**Figures 3A, B**, “PRR sensing and receptor”). We found that compared to NS1(1-126) the NS1(1-126)/ΔPA-X mutant triggered a more pronounced upregulation of TLR3, IRF7, RIG-I, and STAT1 transcription early during the infection (24 hours p.i.), although this difference was not apparent at 48 hours p.i. (**Figures 3A, B**, “PRR sensing and receptor”).

**Figure 4:**
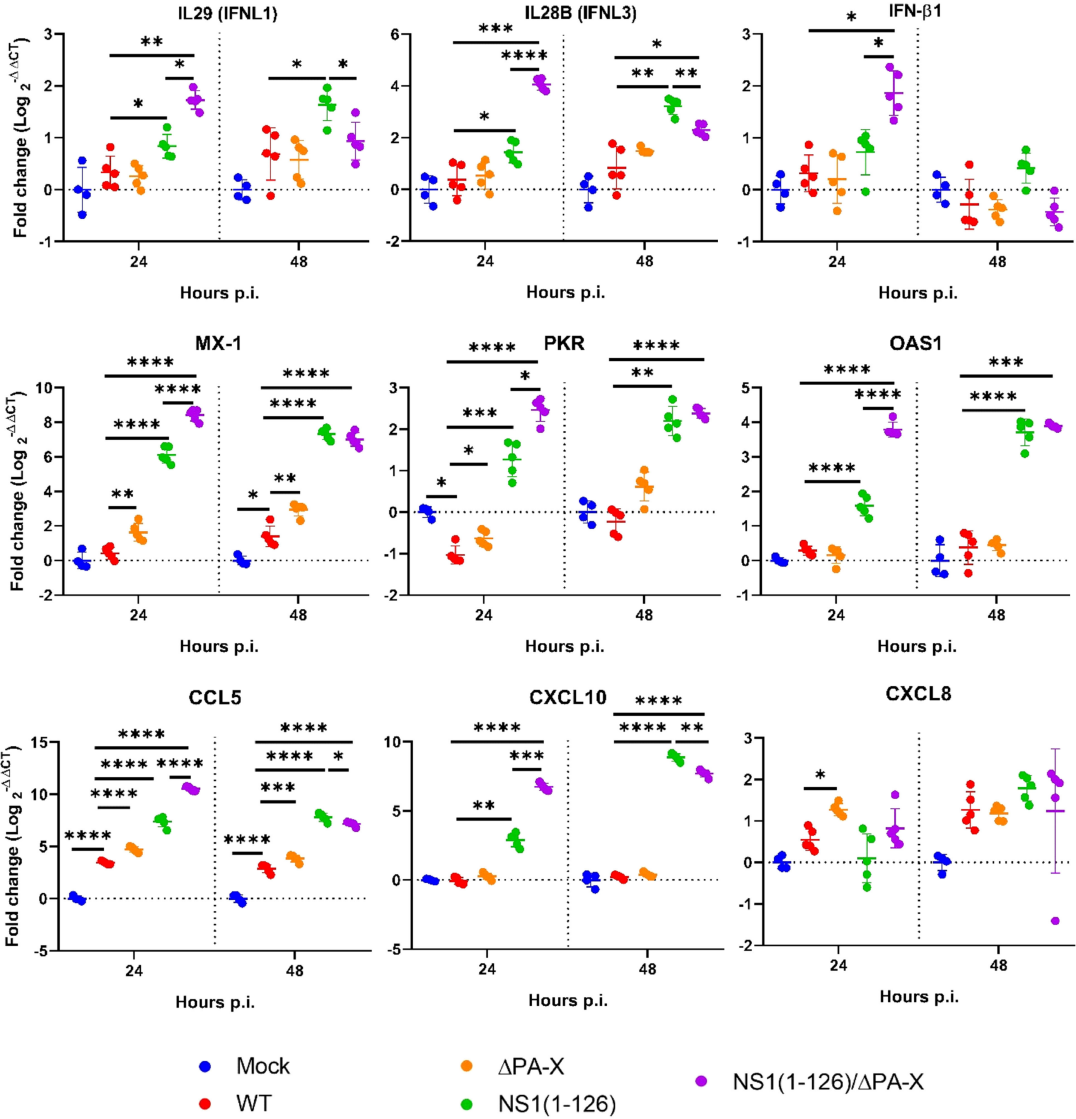
Relative expression levels of selected genes as assessed by RT-qPCR. T3 cells were infected with the indicated viruses using an MOI of 1 TCID_50_/cell. At 24 and 48 hours p.i., real-time RT-qPCR was performed to quantify mRNA levels relative to their expression in mock-infected cells. Significant differences were determined comparing Mock to WT, WT to NS1(1-126), ΔPA-X, or NS1(1-126)/ΔPA-X and NS1(1-126) to NS1(1-126)/ΔPA-X using multiple comparison one-way ANOVA test. *p<0.05, **p<0.01, ***p<0.001, ****p<0.0001 indicate significant difference.

Transcriptomic analysis showed that the IFN response was exclusively triggered by the NS1(1-126) and NS1(1-126)/ΔPA-X mutants (**Figures 3A, B**, “IFN and IFN receptor”, and **C**); moreover, IFN-III appeared to be particularly affected, in accordance with the epithelial characteristics of the T3 cells. Indeed, the expression of IFN-β1 was only transiently upregulated in cells infected with either the NS1(1-126) or the NS1(1-126/ΔPA-X mutant (**Figures 3A, B**, “IFN and IFN receptor”, and **C**). RT-qPCR analysis confirmed the upregulation of IFN-β1 by the NS1(1-126)/ΔPA-X mutant at 24 hours p.i. (**Figure 4**), while transcription of IFNL1 and IFNL3 genes was triggered by both the NS1(1-126) and the NS1(1-126)/ΔPA-X mutant as evident from the transcriptome and RT-qPCR analysis (**Figures 3A, B**, “IFN and IFN receptor”, **C**, **4**). IFN-α was not detected neither by RT-qPCR (**Supplementary Figure 3B**) nor by transcriptomic analysis.

IFNs trigger the expression of interferon-stimulated genes (ISGs), of which several have antiviral functions (e.g., MX1, OAS and PKR), but they also activate the transcription of genes that encode for factors that terminate the IFN response (e.g. SOCS family, USP18). In accordance with the induction of PRR and IFN, infection with NS1(1-126) or NS1(1-126)/ΔPA-X mutants resulted in dramatically high ISG levels while the transcriptome of cells infected with WT pH1N1/09 was quite similar to the transcriptome of mock-infected cells (**Figures 3A, B**, “IFN stimulated genes” and **4**, **Supplementary Tables 2**, **3**). Interestingly, the ΔPA-X mutant also increased expression levels of some ISGs including MX1, PKR and USP18 as evident from both transcriptome analysis and RT-qPCR even though enhanced IFN expression was not observed (**Figure 4**; **Supplementary Figure 3B**). Moreover, the NS1(1-126)/ΔPA-X mutant induced significantly higher levels of some ISGs including IFIT2, MX2, OAS2, IFI44L, and IFIT5, compared to NS1(1-126) at 24 hours p.i. (**Figures 3A, B**, “IFN stimulated genes”). Interestingly, the NS1(1-126)/ΔPA-X mutant induced a faster IFN response in T3 cells compared to the NS1(1-126) mutant with higher levels of mRNA detected by RT-qPCR at 24 hours p.i. However, the IFN response observed for the NS1(1-126)/ΔPA-X mutant at 48 hours p.i. dropped below the response observed for the NS1(1-126) mutant (**Figure 4**).

### 3.4 T3 cells display high chemokine and cytokine expression levels upon infection with the NS1(1-126) and NS1(1-126)/ΔPA-X mutants

IAV infection usually results in the expression of inflammatory and homeostatic cytokines and chemokines that recruit immune cells to the site of infection (Gu et al., 2019). We therefore evaluated the capacity of WT and mutant pH1N1/09 to trigger the expression of cytokines and chemokines in T3 cells. At both 24 and 48 hours p.i., a significantly upregulated transcription of inflammatory cytokines including IL-1α, IL-18 and CXCL8 was detected for the WT virus and the ΔPA-X mutant by transcriptome analysis (**Figures 3A, B**, “cytokine/chemokine”) and by RT-qPCR (**Figure 4**). The NS1(1-126) mutant showed no significant changes compared to mock-treated cells. Interestingly, the NS1(1-126)/ΔPA-X mutant significantly downregulated transcription of these pro-inflammatory cytokines compared to mock-infected cells at 24 hours p.i., but significantly upregulated IL-1α at 48 hours p.i. (**Figures 3A, B**, “cytokine/chemokine”). Moreover, homeostatic cytokines such as IL-33, an alarmin involved in tissue homeostasis and repair, or the hematopoietic growth factor IL-7, were transcriptionally upregulated by the NS1(1-126)/ΔPA-X and NS1(1-126) mutants at 24 hours p.i., while the WT pH1N1/09 virus and the ΔPA-X mutant did not trigger such a response (**Figures 3A, B** “Cytokine/chemokine”).

In addition to cytokines, changes in chemokine levels were also noticed, in particular if the cells were infected with the NS1(1-126) or the NS1(1-126)/ΔPA-X mutant. Indeed, infection with these virus mutants induced enhanced transcription of chemokine genes that are involved in the recruitment of innate immune cells such as CCL5, CXCL8, and CXCL10, both at 24 and 48 hours p.i. (**Figures 3A, B**, “cytokine/chemokine” and **4**). Moreover, the NS1(1-126) and NS1(1-126)/ΔPA-X mutants, but not WT pH1N1/09 and the ΔPA-X mutant, induced higher levels of chemokines involved in the recruitment of lymphocytes such as CXCL10 and CXCL11.

### 3.5 Wild-type and mutant pH1N1/09 display differential effects on gene transcripts involved in cell cycle regulation

**Figure 5:**
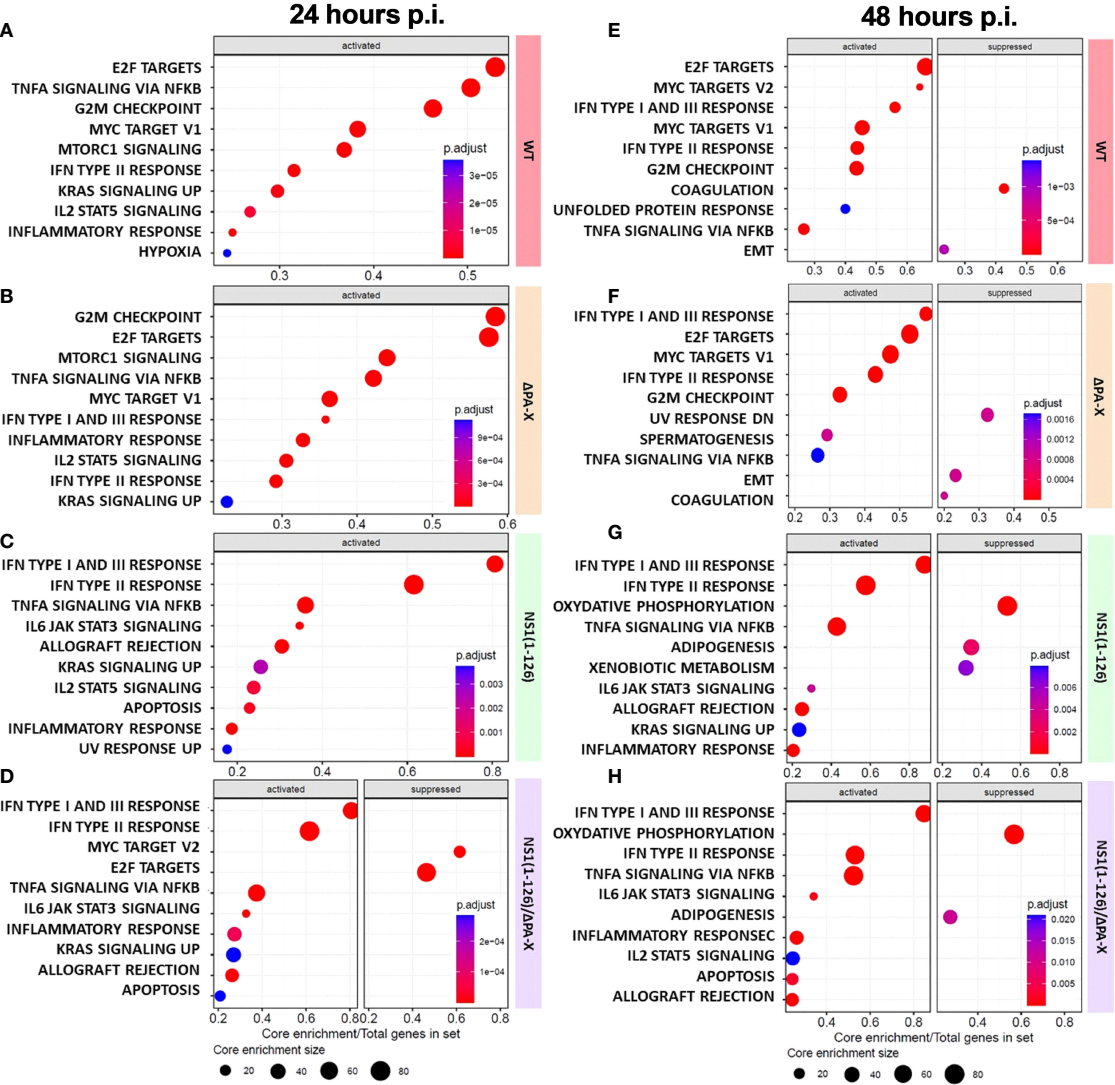
Gene set enrichment analysis (GSEA) of infected compared to mock-treated T3 cells. **(A–H)** GSEA of the 10 most enriched gene sets (activated or suppressed) at 24 hours **(A–D)** and 48 hours **(E–H)** p.i. of T3 cells with the indicated viruses. The enriched gene sets are displayed on the left side and the core enrichment sizes are depicted at the bottom of each panel. The adjusted P-values for each GSEA are represented by the relative size of the dots shown. The viruses used are displayed on the right-hand side of each panel.

To better understand how T3 porcine alveolar epithelial cells respond to infection by WT and mutant pH1N1/09, a gene ontology analysis using the “biological process” gene sets (GO-BP) was performed (**Figure 5**). We observed that infection with either WT virus or ΔPA-X mutant resulted in strong enrichment of gene transcripts encoding proteins involved in cell cycle regulation, such as E2F targets and G2/M checkpoint hallmarks (**Figures 5A, B**). Both E2F targets and G2/M checkpoint gene products ensure and promote correct cell cycle progression and induce apoptosis in damaged cells (Stark and Taylor, 2004; Iaquinta and Lees, 2007). E2F targets and G2M checkpoint remained upregulated for the WT virus and the ΔPA-X at both 24 and 48 hours p.i. (**Figures 5A, B, E, F**). The NS1(1-126) mutant had no impact on E2F targets and G2M checkpoint at both 24 and 48 hours p.i. (**Figures 5C, G**). However, infection with the NS1(1-126)/ΔPA-X mutant led to a significant suppression of E2F targets at 24 hours p.i. (**Figure 5D**). In line with our previous observations (see **Figure 3**), IFN transcripts were only slightly upregulated in cells infected with either the WT pH1N1/09 or the ΔPA-X mutant. In contrast, the strongest IFN transcripts upregulation was found in NS1(1-126) and NS1(1-126)/ΔPA-X infected cells, at both time points (**Figure 5**). Interestingly, the GO-BP “inflammatory response” was activated by all four viruses. In addition to that, the gene transcripts involved in oxidative phosphorylation were highly downregulated in T3 cells at 48 hours p.i. with either the NS1(1-126)/ΔPA-X or the NS1(1-126) mutant (**Figures 5G, H**). The ability to suppress oxidative phosphorylation is intriguing since an upregulation was reported to be beneficial for influenza replication (Foo et al., 2022). Overall, these data indicate that infection of T3 cells by WT pH1N1/09 virus or the ΔPA-X mutant resulted in enhanced transcription of genes involved in cell cycle progression while NS1(1-126)/ΔPA-X mutants lost this capacity.

**Figure 6:**
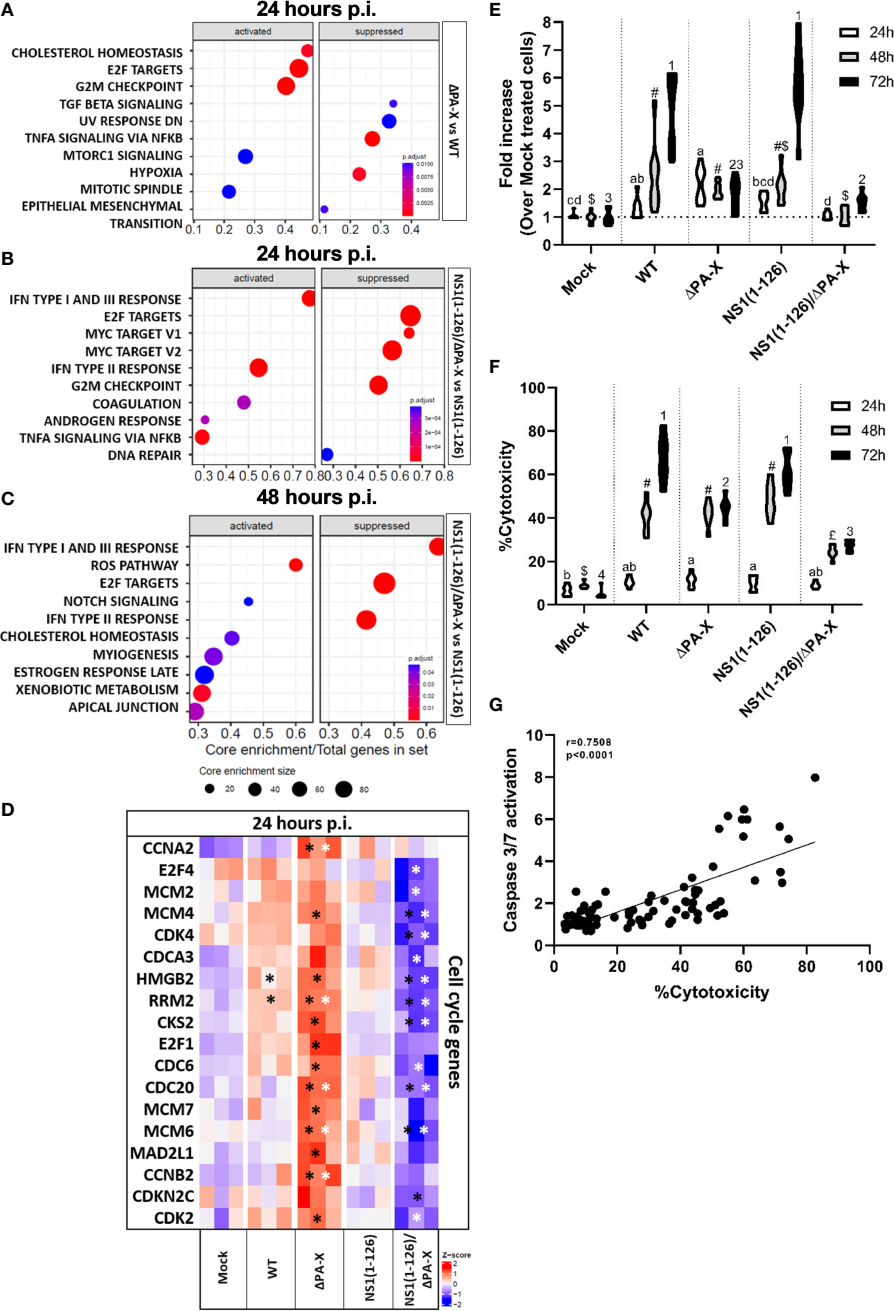
Comparison of ΔPA-X to WT and NS1(1-126)/ΔPA-X to NS1(1-126) virus-infected T3 cells. **(A)** GSEA of the 10 most enriched gene sets in T3 cells infected with ΔPA-X compared to WT 24 hours p.i. **(B)** GSEA of the 10 most enriched pathways in T3 cells infected with NS1(1-126)/ΔPA-X compared to NS1(1-126) at 24 hours p.i. **(C)** GSEA of the 10 most enriched pathways in T3 cells infected with NS1(1-126)/ΔPA-X compared to NS1(1-126) at 48 hours p.i. **(D)** Heatmaps illustrating the relative expression levels of important differentially expressed cell cycle genes in T3 cells 24 hours p.i. with the indicated viruses. Genes from infected cells showing significant differences to mock-infected cells are indicated by black stars while genes of ΔPA-X and NS1(1-126)/ΔPA-X virus-infected cells showing significant differences compared to WT and NS1(1-126) virus-infected cells, respectively, are indicated by white stars. The Z-score indicates upregulated (red color) or downregulated gene expression (blue color). The adjusted p-value of each comparison is presented in **Supplementary Table 4**. **(E)** Detection of caspase-3/7 activity in T3 cells at 24, 48, and 72 hours p.i. with the indicated viruses. **(F)** Detection of LDH release in the supernatant in T3 cells at 24, 48, and 72 hours p.i. with the indicated virus. For **(E, F)**, conditions showing no significant difference at 24 hours p.i. are marked by the same letter, no significant difference at 48 hours p.i. are marked by the same symbol, and no difference at 72 hours p.i. are marked by the same number. Activation of caspase 3/7 and LDH release was correlated **(G)**. Significant differences were assessed using a multiple comparison two-way ANOVA test **(E, F)** and Spearman’s correlation **(G)**. Genes from infected cells showing significant differences to mock-infected cells are indicated by *.

To better understand how ΔPA-X mutant modulates the host response compared to WT virus, and how the response induced by the NS1(1-126) mutant was modulated in the absence of the PA-X protein, we analyzed the top 10 gene set enrichments of cells infected with either the ΔPA-X or the NS1(1-126)/ΔPA-X mutant compared to cells infected by either WT virus or NS1(1-126) mutant respectively (**Figures 6A–C**). Strikingly, even though both WT virus and ΔPAX mutant induced the upregulation of cell cycle gene transcripts. (**Figures 5A, B, E, F**), the ΔPA-X mutant additionally enhanced the transcription of E2F targets and G2/M checkpoints at 24 hours p.i. (**Figure 6A**), which also induced a strong upregulation of cyclin-dependent kinase (CDK), G1/S and G2/mitotic-specific cyclin (CCN), and cell cycle gene (CDC) (**Figure 6D**). Notably, top 10 genes set enrichment revealed that the inflammatory response (TNF-α signaling via NFκB) was also suppressed in the absence of PA-X. Interestingly, cholesterol homeostasis gene sets were upregulated (**Figure 6A**), suggesting that PA-X plays a role in regulating steroid biogenesis, a phenomenon previously observed for influenza A virus infection (Bai et al., 2022) but not yet linked to PA-X. Of note, no changes were observed for the ΔPA-X mutant compared to WT pH1N1/09 at 48 hours p.i. (**Supplementary Figure 7**).

Interestingly, avoiding PA-X expression in the NS1(1-126) background caused different gene set enrichments when compared to virus with fully functional NS1 (**Figures 6B, C**). Infection with the NS1(1-126)/ΔPA-X mutant caused a pronounced downregulation of cell cycle gene transcripts compared to the NS1(1-126) mutant, and this response was still present at 48 hours p.i. (**Figures 6B, C**). CDK, CDC and CNN gene family transcripts were significantly more downregulated by the NS1(1-126)/ΔPA-X mutant compared to the NS1(1-126) mutant (**Figure 6D**; **Supplementary Table 4**).

Cell cycle disturbance is often associated with activation of apoptosis (Pucci et al., 2000). To figure out whether PA-X triggers apoptosis or not, caspase 3/7 activity was measured at 24, 48, and 72 hours p.i. with the WT and mutant viruses (**Figure 6E**). Infection of T3 cells with WT pH1N1/09 resulted in increasing levels of active caspase 3/7 from 24 to 72 hours p.i. Interestingly, the ΔPA-X mutant induced a weak caspase 3/7 activation along the experiment, which might be due to a downstream effect of disturbance of cell cycle regulation. Moreover, NS1(1-126) did not show any difference compared to WT pH1N1/09 with increasing caspase 3/7 activity over time. Even though infection with the NS1(1-126)/ΔPA-X mutant resulted in reduced transcription of cell cycle genes, this mutant induced a very weak and late apoptosis activation, that it was lower to this induced by the ΔPA-X mutant. To confirm that the activation of caspase 3/7 induce apoptosis, release of lactate dehydrogenase (LDH) in the supernatant was measured at 24, 48 and 72 hours p.i. with the WT and mutant viruses (**Figure 6F**). Increasing level of LDH was measured for WT and NS1(1-126) from 24 to 72 hours p.i., while LDH release increase between 24 et 48 hours p.i. but remained at the same level between 48 and 72 hours p.i. for ΔPA-X and NS1(1-126)/ΔPA-X. Activation of caspase 3/7 and LDH release upon infection with WT and mutant viruses show strong correlation over time (**Figure 6G**).

## 4 Discussion

IAV with modified NS1 genes has long been considered as potential LAIV candidates (Nogales et al., 2022). However, it turned out that it is extremely difficult to achieve an appropriate balance between attenuation and safety on one hand and immunogenicity and vaccine efficacy on the other hand (McLean and Belongia, 2021). In addition to the NS1 protein, the recently discovered accessory PA-X protein was also shown to exhibit host shut-off activity, and therefore represents an attractive target for the generation of LAIV (Hayashi et al., 2015; Nogales et al., 2018; Hilimire et al., 2020; Dunagan et al., 2021).

In the present work, we took advantage of reverse genetics to generate viruses with a truncated NS1(1-126) protein and/or a knockout of the PA-X protein that were based on a swine-origin human pandemic influenza virus isolate, A/Hamburg/4/2009 (H1N1), and investigated the response of a recently established T3 porcine bronchiolar epithelial cell line to infection with these viruses. We found that infection of T3 cells with pH1N1/09 WT virus resulted in a moderate suppression of host gene expression including type I and type III IFNs. In line with the absence of IFN, the Janus kinase inhibitor ruxolitinib had no effect on WT virus replication.

The ΔPA-X virus showed a very similar replication kinetics as WT virus, and like WT virus, was not affected by ruxolitinib. In line with this phenotype, infection with ΔPA-X virus did not result in the upregulation of IFNs and ISGs expression. However, in contrast to WT virus a significant increase in the transcription of cell cycle-related genes was observed at 24 hours p.i. Moreover, ΔPA-X virus did not trigger apoptosis to the same extent as WT virus, although both viruses replicated to similar titers. This suggests that the PA-X protein of pH1N1/09 does not cause a global host shut-off but may specifically target only certain genes. Our findings seem to contradict previous works showing that PA-X is a key factor that mediates a global host shut-off (Hayashi et al., 2015; Gaucherand et al., 2019; Ma et al., 2019). However, this discrepancy might be explained by the fact that pH1N1/09 expresses a C-terminally truncated PA-X protein of 232 amino acids (Shi et al., 2012), whereas most IAV encode PA-X proteins of 252 amino acids. The missing C-terminal region of 20 amino acids is known to contribute to PA-X host shutoff activity (Bavagnoli et al., 2015; Gao et al., 2015; Hu et al., 2018). Indeed, apart from pandemic 2009 H1N1 viruses, equine H7N7, canine H3N8, canine H3N2 and bat influenza viruses, show a truncation of the PA-X C-terminal domain, which most likely represents an adaptation to their hosts. The truncated C-terminus of the PA-X protein might explain the little impact the ΔPA-X virus had on the transcription of most genes in infected T3 cells. However, loss of PA-X in the related A/California/04/2009 (H1N1) has been reported to cause a reduction in viral replication and pathogenicity as well as increased innate immune responses in mice (Gao et al., 2015; Hayashi et al., 2015; Lee et al., 2017). Nevertheless, it should also be considered that the PA-X protein may mediate host shut-off activity in a species- and/or cell type-specific manner.

In contrast to WT and ΔPA-X virus, infection of T3 cells with NS1(1-126) virus triggered the transcription of various host factors including IFN genes and ISGs. Consequently, this virus replicated to lower titers on these cells and the attenuation was overcome by the Janus kinase inhibitor ruxolitinib, indicating that the attenuation of the virus was at least in part due to the induction of IFN. Interestingly, the corresponding NS1(1-126) mutant of TX/98 was found to replicate on porcine PK15 cells in a much more attenuated manner than our pH1N1/09 based NS1(1-126) mutant (Solórzano et al., 2005). This discrepancy could be a result of cell-type specific differences between PK15 and T3 cells, but might also be a consequence of the different IAV strains used. It should be noted that the NS1 protein of the pH1N1/09 WT virus is characterized by an 11 amino acid truncation, which has been linked to inefficient inhibition of host gene expression without affecting virus replication (Tu et al., 2011). In addition, the 2009 pandemic H1N1 IAV NS1 protein has been shown to be unable to block general host gene expression in human or porcine cells due to low binding to cellular pre-mRNA processing protein CPSF30 (Hale et al., 2010).

In contrast to the NS1(1-126) mutant, the NS1(1-126)/ΔPA-X double mutant was clearly attenuated in T3 cells. The attenuated replication of NS1(1-126)/ΔPA-X was partially restored by ruxolitinib, indicating that the attenuation was only partially mediated by IFN. In line with this observation, the NS1(1-126)/ΔPA-X double mutant induced expression of IFNs and ISGs at higher levels than the NS1(1-126) mutant early in infection (24 hours p.i., see **Figure 4**). However, in contrast to NS1(1-126) virus, the NS1(1-126)/ΔPA-X double mutant strongly suppressed the transcription of genes that are involved in cell cycle regulation. The reasons for this synergistic effect are not known but it might be speculated that there is a cross-talk between cell cycle regulators (suppressed by PA-X) and components of the innate immune system (suppressed by NS1). It seemed that upregulation of cell cycle gene expression was accompanied by downregulation of proapoptotic factors and vice versa, suppression of cell cycle genes resulted in the upregulated of proapoptotic genes (see **Figure 6**). However, caspase-3/7 activity, a key initiator caspase of the apoptotic pathway, remained low in NS1(1-126)/ΔPA-X infected T3 cells for the whole 72 hours of infection time. Either the virus did not trigger apoptosis (in contrast to WT virus) or it actively suppressed it. Caspase-3 activation has been shown to be essential for efficient IAV propagation (Wurzer et al., 2003) as it results in enlargement of the nuclear pores which promotes nuclear export of viral ribonucleoprotein complexes late in the infection cycle (Mühlbauer et al., 2015). However, no correlation between caspase 3/7 activity and virus replication efficacy was observed as the ΔPA-X virus replicated as efficiently as WT virus although caspase-3/7 activity was reduced. It is not excluded that apoptosis inhibition together with elevated IFN- I and -III response might be necessary to reduce virus replication as seen for NS1(1-126)/ΔPA-X in T3 cells. As cells infected with either the ΔPA-X or the NS1(1-126)/ΔPA-X mutant showed low caspase-3/7 activity, it is likely that the absence of PA-X-mediated host shut-off activity is responsible for this phenomenon. Future studies should focus on the mechanisms of how PA-X interferes with apoptosis regulation.

In conclusion, our findings demonstrate that NS1 and PA-X act in a concerted manner to modulate the host response thereby promoting viral replication. The NS1(1-126)/ΔPA-X double mutant might represent an attractive LAIV candidate. It not only showed attenuated virus replication in a bronchiolar epithelial cell line and a human type II pneumocyte-derived tumor cell line but also induced a cocktail of cytokines and chemokines that may stimulate the immune response. In particular, type III IFN was identified as a potent mucosal adjuvant that was demonstrated to strongly promote mucosal immunity against influenza virus infection in a mouse model (Klinkhammer et al., 2018; Ye et al., 2019). Moreover, the induction of IFN will not only restrict LAIV infection but will also prevent co-infection with IAV field strains thereby reducing the risk that reassortant viruses will emerge. The lack of apoptosis in NS1(1-126)/ΔPA-X virus-infected cells is another plus, since this will reduce LAIV-induced cell death and tissue damage in the respiratory tract. *In vivo* studies are currently performed in order to primarily evaluate the efficacy and safety of these potential swine LAIV vaccine in the pig model but also to assess if potential translation of such LAIV vaccine to human would be conceivable.

## Data availability statement

The data presented in the study are deposited in the ENA arrayexpress repository, accession number E-MTAB-13128.

## Author contributions

RA acquired and analyzed data, and wrote the manuscript. GZ and AS conceived the idea, designed the study and reviewed the manuscript. OG-N helped with interpretation of the data and reviewed the manuscript. All authors gave their final approval for submission.
