# Supplementary material for: NS1 and PA-X of H1N1/09 influenza virus act in a concerted manner to manipulate the innate immune response of porcine respiratory epithelial cells
Source: Front Cell Infect Microbiol. 2023 Jul 26;13:1222805. doi: 10.3389/fcimb.2023.1222805 (PMC10410561; doi:10.3389/fcimb.2023.1222805)
Supplement: Supplementary file 1 [file DataSheet_1.docx]

Supplementary Material

NS1 and PA-X of H1N1/09 influenza virus act in a concerted manner to manipulate the innate immune response of porcine respiratory epithelial cells

**Robin Avanthay^1,2,3^, Obdulio Garcia-Nicolas^1,2^, Gert Zimmer^1,2^, Artur Summerfield^1,2^**

^1^ Institute of Virology and Immunology, Mittelhäusern, Switzerland.
^2^ Department of Infectious Diseases and Pathobiology, Vetsuisse Faculty, University of Bern, Bern, Switzerland.
^3^ Graduate School for Cellular and Biomedical Sciences, University of Bern, Bern, Switzerland.

# Supplementary Figures and Tables

## Supplementary Figure 1
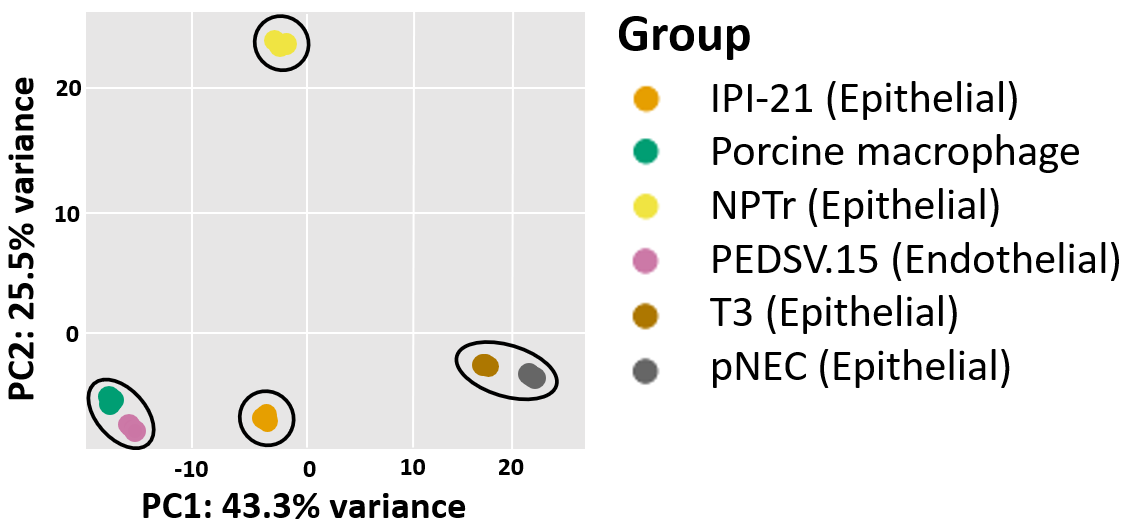
Supplementary Figure 1. PCA analysis of the transcriptome of six different porcine cell types including macrophages, intestinal epithelial cell line (IPI-21), tracheal epithelial cell line (NPTr), endothelial cell line (PEDSV.15), nasal epithelial primary basal cells (pNEC), and T3 cells.

## Supplementary Figure 2


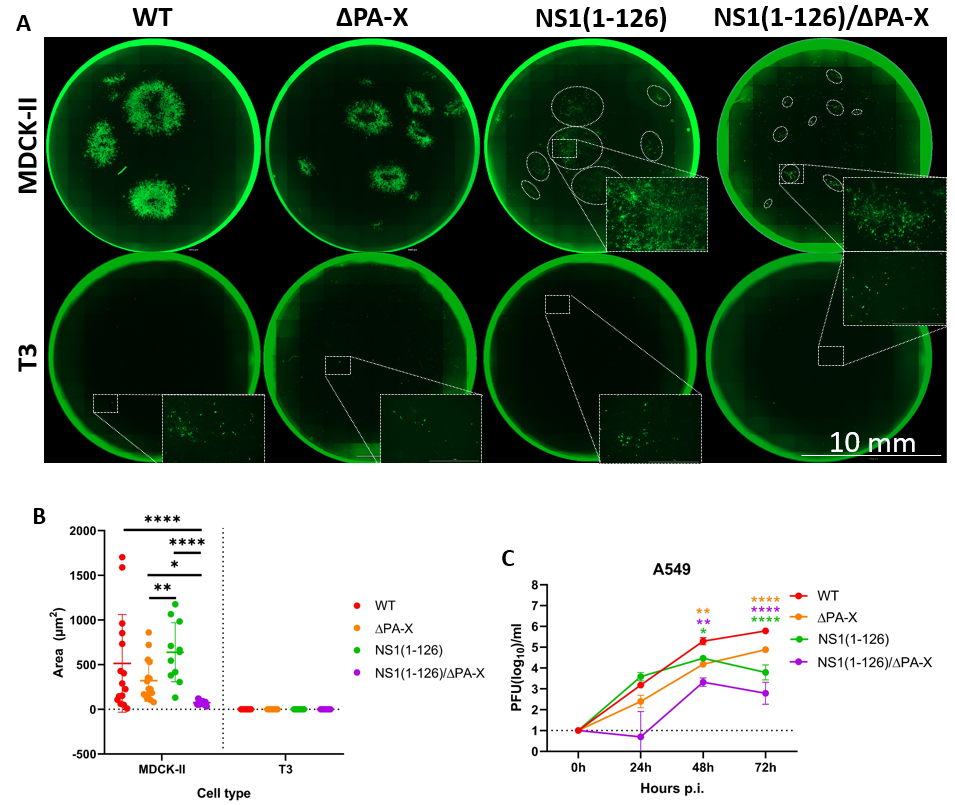


**Supplementary Figure 2.** (A) Plaque formation in MDCK-II and T3 cells 72 hours following infection with the indicated viruses. Infected cells were detected by indirect immunofluorescence using a monoclonal antibody directed to the NP antigen (green fluorescence). Boxed areas show fields at 10x magnification. Plaque formation in MDCK-II cells infected with NS1(1-126) and NS1(1-126)/ΔPA-X are encircled in white. (B) Determination of plaque size in MDCK-II and T3 cells in µm^2^. Significant differences in plaque size as calculated by the one-way ANOVA test are indicated. (C) A549 cells were infected with the indicated viruses using an MOI of 0.0001 TCID_50_/cell and maintained at 37°C. At the indicated times, cell culture supernatant was collected and infectious virus titers determined. Significant differences between WT and NS1(1-126), ΔPA-X, or NS1(1-126)/ΔPA-X were determined using two-way ANOVA test. *p<0.05, **p<0.01, ***p<0.001, ****p<0.0001 indicate significant differences.

## Supplementary Figure 3


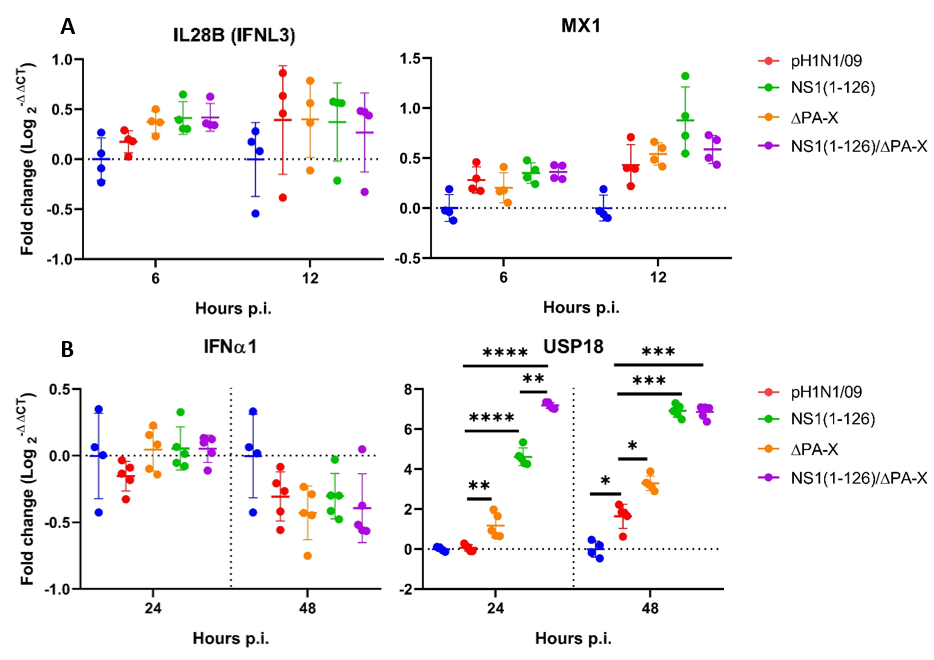


**Supplementary Figure 3.** T3 cells were infected with the indicated viruses using an MOI of 1 TCID_50_/cell. At the indicated times post infection, real-time RT-qPCR was performed to quantify mRNA levels relative to their expression in mock-treated cells. Significant differences were determined comparing mock to WT, WT to NS1(1-126), ΔPA-X, or NS1(1-126)/ΔPA-X and NS1(1-126) to NS1(1-126)/ΔPA-X using multiple comparison two-way ANOVA test. *p<0.05, **p<0.01, ***p<0.001, ****p<0.0001 indicate significant difference.

## Supplementary Figure 4


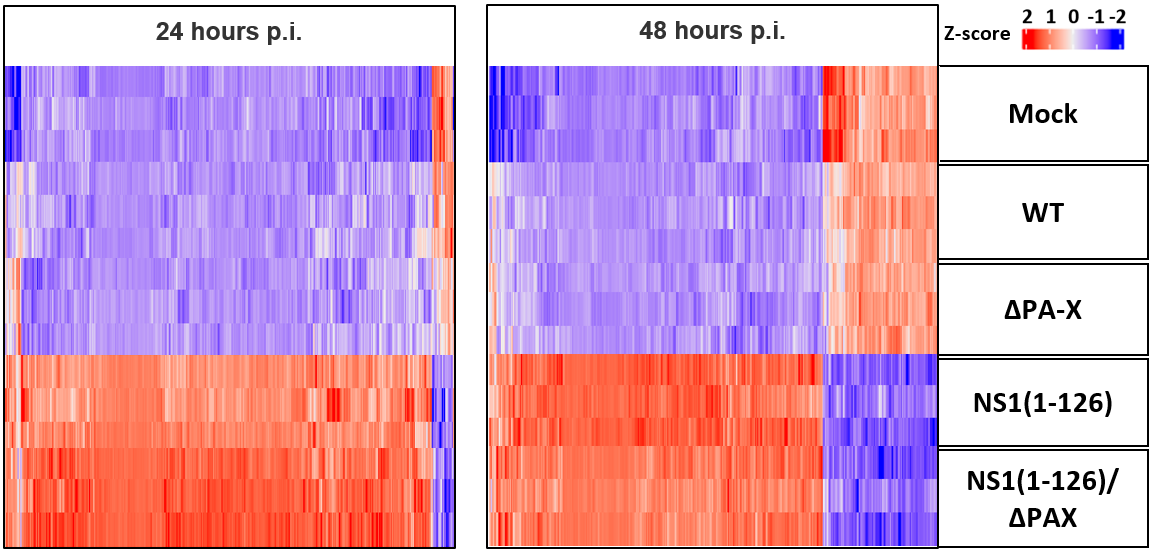


**Supplementary Figure 4.** Top 500 DEGs with significantly changes of expression levels 24 and 48 hours p.i. of T3 cells with the indicated viruses.

## Supplementary Figure 5


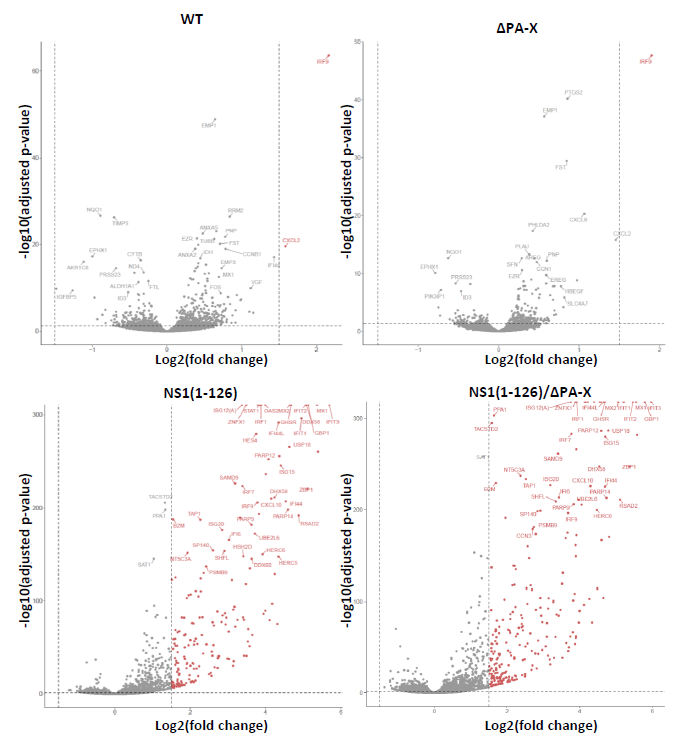


**Supplementary Figure 5.** Volcano plot showing the most prominent DEGs 24 hours p.i. of T3 cells with the indicated viruses. The y-axis represents the adjusted p-value (-log_10_). The x-axis represents the fold-change (log_2_) of gene expression. The fold-change threshold was set to 1.5.

## Supplementary Figure 6


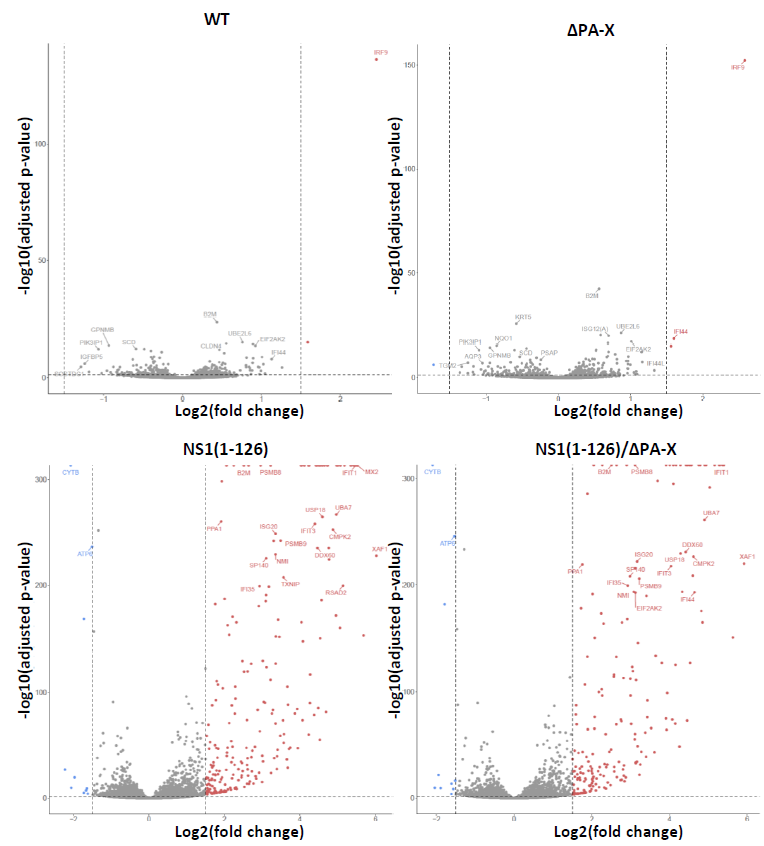


**Supplementary Figure 6.** Volcano plot showing the most prominent DEGs 48 hours p.i. of T3 cells with the indicated viruses. The y-axis represents the adjusted p-value (-log_10_). The x-axis represents the fold-change (log_2_) of gene expression. The fold-change threshold was set to 1.5.

## Supplementary Figure 7


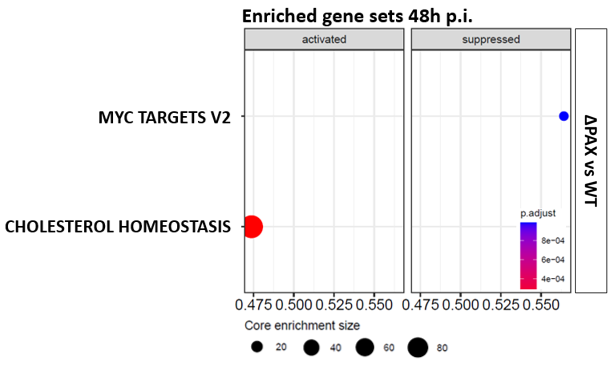


**Supplementary Figure 7.** Gene set enrichment analysis of T3 cells infected with ΔPA-X compared to WT virus. GSEA of the 10 most enriched gene sets in T3 cells 48 hours p.i. with ΔPA-X compared to WT virus. The enriched pathways are displayed on the left side and the core enrichment sizes on the bottom of each panel. The adjusted P-values of each GSEA are represented by the dot sizes plotted in the diagram.

## Supplementary Table 1. List of oligonucleotide primers and probes.

| Gene | Primer / probe | Sequence | Concentration (nm) | References |
| --- | --- | --- | --- | --- |
| 18S | Forward | CGCCGCTAGAGGTGAAATTC | 400 | (Alves et al., 2009) |
|  | Reverse | GGCAAATGCTTTCGCTCTG | 400 |  |
|  | Probe | TGGACCGGCGCAAGACGGA | 100 |  |
| IFN-λ1 | Forward | CAGGCTTGCATCAGGGCTCA | 400 | This study |
|  | Reverse | GGTGCAGCCAGTGGTTGAGG | 400 |  |
|  | Probe | CCCACGGCAGGATCCCGGCT | 100 |  |
| IFN-λ3 | Forward | GAGCTGCCTGGAAGCCTCTG | 400 | This study |
|  | Reverse | CATCCAAGACGCTGGGACAGG | 400 |  |
|  | Probe | CCGCCTCCTCACCCGGGACC | 100 |  |
| IFN-β1 | Forward | GGCTGGAATGAAACCGTCAT | 400 | This study |
|  | Reverse | TCCAGGATTGTCTCCAGGTCA | 400 |  |
|  | Probe | CCTTGTGGAACTTGATGGGCAGATGG | 100 |  |
| IFN-α1 | Forward | GGCTGGAATGAAACCGTCAT | 400 | This study |
|  | Reverse | TCCAGGATTGTCTCCAGGTCA | 400 |  |
|  | Probe | CCTTGTGGAACTTGATGGGCAGATGG | 100 |  |
| MX-1 | Forward | CAGCACCTGATTGCCTACCA | 400 | This study |
|  | Reverse | GGTCCGGAGGATGAAGAACTG | 400 |  |
|  | Probe | AGGCGCATCTCCAGCCACATCCCT | 100 |  |
| PKR | Forward | AGCAGCTCTCCCACAACGA | 400 | This study |
|  | Reverse | GACGTATTTGCTGAGAAGCCATT | 400 |  |
|  | Probe | CACATCGGCTTCAGAGTCAGCAACTGA | 100 |  |
| OAS1 | Forward | GCGCCGAGGAGAATTCATC | 400 | This study |
|  | Reverse | TGGACCTCAAACGTCACTTTAAAC | 400 |  |
|  | Probe | CTCTTTGACAGGCTTCCAGCTGTCTCC | 100 |  |
| CCL5 | Forward | TCCATGGCAGCAGTCGTCTT | 400 | This study |
|  | Reverse | CAGGCTCAAGGCTTCCTCCA | 400 |  |
|  | Probe | ACCGCCAGGTGTGTGCCAACCCAGA | 100 |  |
| CXCL10 | Forward | TTGAAATGATTCCTGCAAGTCAA | 400 | (Moraes et al., 2007) |
|  | Reverse | GACATCTTTTCTCCCCATTCTTTT | 400 |  |
|  | Probe | CTTGCCCACATGTTGAGATCATTGCCAC | 100 |  |
| IL8 | Forward | CCGTGTCAACATGACTTCCAA | 400 | (Arceo et al., 2013) |
|  | Reverse | GCCTCACAGAGAGCTGCAGAA | 400 |  |
|  | Probe | TTCTTCGCCCTCAGTGTGAA | 100 |  |

## Supplementary Table 2. Summary statistics of immune gene transcripts presented in Fig. 3A

Adjusted p-value of differentially expressed immune genes in cells 24 hours p.i. with either WT, ΔPA-X, NS1(1-126), and NS1(1-126)/ΔPA-X compared to Mock-treated cells, in ΔPA-X infected cells compared to WT virus infected cells, and in NS1(1-126)/ΔPA-X compared to NS1(1-126) infected cells.

| Gene name  24h post infection | WT | | ΔPA-X | NS1(1-126) | NS1(1-126)/ ΔPA-X | ΔPA-X vs WT | NS1(1-126)/ΔPA-X vs NSΔ93 |
| --- | --- | --- | --- | --- | --- | --- | --- |
| MYD88 | | ns | ns | 9.23E-13 | 1.69E-20 | ns | ns |
| TLR3 | | ns | ns | 3.45E-39 | 1.14E-68 | ns | 1.36E-03 |
| STAT2 | | ns | ns | 2.85E-41 | 1.18E-51 | ns | ns |
| TRIM25 | | ns | ns | 5.78E-73 | 2.16E-85 | ns | ns |
| IRF7 | | ns | ns | 3.11E-224 | 3.36E-284 | ns | 1.54E-03 |
| RIG-I (DDX58) | | ns | 5.11E-07 | <9.99E-299 | <9.99E-299 | ns | 2.84E-03 |
| STAT1 | | ns | 7.27E-04 | <9.99E-299 | <9.99E-299 | ns | 1.36E-02 |
| IFIH1 | | ns | 3.06E-02 | 1.64E-80 | 1.28E-86 | ns | ns |
| IRF1 | | ns | ns | <9.99E-299 | <9.99E-299 | ns | ns |
| IFNGR2 | | ns | ns | ns | ns | ns | ns |
| IFNAR1 | | ns | 3E-02 | 2.29E-10 | 2.55E-16 | ns | ns |
| IFNB1 | | ns | ns | 6.19E-06 | 6.19E-06 | ns | ns |
| IFNAR2 | | ns | ns | 1.13E-08 | 1.13E-08 | ns | ns |
| IFNL1 (IL29) | | ns | ns | 1.41E-02 | 2.08E-02 | ns | ns |
| OAS1 | | ns | ns | 8.16E-42 | 3.51E-54 | ns | ns |
| OASL | | ns | ns | 8.77E-119 | 1.2E-149 | ns | ns |
| ISG15 | | ns | 1.27E-02 | 2.86E-246 | 2.47E-281 | ns | ns |
| IFIT2 | | ns | 3.25E-04 | <9.99E-299 | <9.99E-299 | ns | 2.03E-06 |
| IFIT3 | | ns | 7.7E-04 | <9.99E-299 | <9.99E-299 | ns | ns |
| MX2 | | ns | ns | <9.99E-299 | <9.99E-299 | ns | 2.4E-05 |
| OAS2 | | ns | ns | <9.99E-299 | <9.99E-299 | ns | 2.92E-04 |
| SOCS1 | | ns | ns | 9.44E-98 | 1.17E-144 | ns | ns |
| IFI44L | | ns | 1.31E-02 | 1.4E-292 | <9.99E-299 | ns | 4.19E-02 |
| IFIT5 | | ns | ns | 2.41E-89 | 1.07E-104 | ns | ns |
| ISG20 | | ns | ns | 6.29E-177 | 1.33E-227 | ns | 4.71E-03 |
| PKR | | ns | 8.34E-03 | 9.48E-83 | 3.9E-93 | ns | ns |
| MX1 | | 5.37E-09 | 2.74E-13 | <9.99E-299 | <9.99E-299 | ns | 2.26E-2 |
| USP18 | | 2.56E-04 | 5.11E-07 | 1.61E-266 | 1.35E-287 | ns | ns |
| ADAR | | ns | ns | 4.16E-60 | 5.29E-72 | ns | ns |
| SOCS3 | | ns | ns | 1.14E-03 | 2.16E-02 | ns | ns |
| IL18 | | ns | ns | ns | 1.17E-02 | ns | ns |
| IL1A | | 1.21E-02 | ns | ns | 4.84E-04 | ns | ns |
| CXCL8 | | 4.97E-21 | 5.55E-05 | ns | 1.93E-08 | ns | 1.24E-02 |
| CCL5 | | ns | ns | 2.42E-07 | 2.43E-05 | ns | ns |
| CXCL10 | | ns | ns | 8.66E-211 | 1.10E-226 | ns | ns |
| CXCL11 | | ns | ns | 5.02E-39 | 1.9E-43 | ns | ns |
| IL7 | | ns | ns | 1.54E-14 | ns | ns | ns |
| IL33 | | ns | ns | 2.87E-02 | 3.32E-03 | ns | ns |

## Supplementary Table 3. Summary statistics of immune gene transcripts presented in Fig. 3B

Adjusted p-value of differentially expressed immune genes in cells 48 hours p.i. with either WT, ΔPA-X, NS1(1-126), or NS1(1-126)/ΔPA-X compared to Mock-treated cells, in ΔPA-X infected cells compared to WT virus infected cells, and in NS1(1-126)/ΔPA-X compared to NS1(1-126) infected cells.

| Gene name  48h post infection | | WT | ΔPA-X | NS1(1-126) | NS1(1-126)-ΔPA-X | ΔPA-X vs WT | NS1(1-126)/ΔPA-X vs NSΔ93 |
| --- | --- | --- | --- | --- | --- | --- | --- |
| MYD88 | ns | | ns | 3.94E-4 | ns | ns | ns |
| TLR3 | ns | | ns | 7.21E-32 | 2.22E-26 | ns | ns |
| STAT2 | ns | | ns | 5.67E-55 | 8.52E-40 | ns | ns |
| TRIM25 | ns | | ns | 2.18E-32 | 1.4E-19 | ns | ns |
| IRF7 | ns | | ns | <9.99E-299 | <9.99E-299 | ns | ns |
| RIG-I (DDX58) | 2.42E-2 | | ns | 7.31E-51 | 2.59E-126 | ns | ns |
| STAT1 | ns | | ns | 2.2E-168 | 6.52E-146 | ns | ns |
| IFIH1 | 2.01E-5 | | 1.45E-3 | 7.39E-92 | 1.4E-19 | ns | ns |
| IRF1 | ns | | ns | 8.61E-50 | 1.19E-30 | ns | ns |
| IFNGR2 | ns | | ns | 1.07E-15 | 2.45E-28 | ns | ns |
| IFNAR1 | ns | | ns | ns | ns | ns | ns |
| IFNB1 | ns | | ns | ns | ns | ns | ns |
| IFNAR2 | ns | | ns | 4.8E-3 | ns | ns | ns |
| IFNL1 (IL29) | ns | | ns | ns | ns | ns | ns |
| OAS1 | ns | | ns | 2.9E-56 | 1.2E-49 | ns | ns |
| OASL | ns | | ns | <9.99E-299 | <9.99E-299 | ns | ns |
| ISG15 | ns | | ns | <9.99E-299 | <9.99E-299 | ns | ns |
| IFIT2 | ns | | ns | 4.77E-166 | 9.22E-135 | ns | ns |
| IFIT3 | ns | | ns | 5.81E-259 | 3.02E-218 | ns | ns |
| MX2 | ns | | ns | <9.99E-299 | <9.99E-299 | ns | ns |
| OAS2 | ns | | ns | <9.99E-299 | <9.99E-299 | ns | ns |
| SOCS1 | ns | | ns | 4.47E-5 | 2.6E-2 | ns | ns |
| IFI44L | 1.8E-5 | | 2.69E-8 | 1.89E-172 | 2.31E-165 | ns | ns |
| IFIT5 | 5.43E-5 | | ns | 2.83E-84 | 1.93E-70 | ns | ns |
| ISG20 | ns | | ns | 1.01E-249 | 1.43E-22 | ns | ns |
| PKR | 1.87E-14 | | ns | 1.8E-199 | 2.11E-193 | ns | ns |
| MX1 | 1.35E-2 | | 1.2E-4 | <9.99E-299 | <9.99E-299 | ns | 2.26E-2 |
| USP18 | 3.89E-6 | | 8.77E-6 | 1.59E-295 | 3.45E-230 | ns | ns |
| ADAR | ns | | ns | 1.96E-88 | 7.29E-66 | ns | ns |
| SOCS3 | ns | | ns | ns | ns | ns | ns |
| IL18 | ns | | ns | ns | ns | ns | ns |
| IL1A | 3.72E-2 | | ns | ns | 2.1E-2 | ns | ns |
| CXCL8 | 6.13E-4 | | 3.85E-5 | ns | ns | ns | ns |
| CCL5 | ns | | ns | 3.73E-20 | 2.06E-11 | ns | ns |
| CXCL10 | ns | | ns | 9.73E-62 | 4.04E-35 | ns | 1.51E-02 |
| CXCL11 | ns | | ns | 4.27E-85 | 1.14E-62 | ns | ns |
| IL7 | ns | | ns | 2.75E-2 | ns | ns | ns |
| IL33 | ns | | ns | ns | ns | ns | ns |

## Supplementary Table 4. Summary statistics of immune gene transcripts presented in Fig. 6D

Adjusted p-value of differentially expressed cell cycle genes in cells 24 hours p.i. with either WT, ΔPA-X, NS1(1-126), or NS1(1-126)/ΔPA-X compared to Mock-treated cells, in ΔPA-X infected cells compared to WT virus infected cells, and in NS1(1-126)/ΔPA-X infected cells compared to NS1(1-126) infected cells.

| Gene name  24h post infection | | WT | ΔPAX | NS1(1-126) | NS1(1-126)/ ΔPA-X | ΔPA-X vs WT | NS1(1-126)/ΔPA-X vs NS1(1-126) |
| --- | --- | --- | --- | --- | --- | --- | --- |
| CCNA2 | ns | | 1.26E-08 | ns | ns | 2.25E-04 | ns |
| E2F4 | ns | | ns | ns | ns | ns | 1.79E-03 |
| MCM2 | ns | | ns | ns | ns | ns | 9.98E-04 |
| MCM4 | ns | | 3.52E-03 | ns | 1.07E-05 | ns | 3.76E-03 |
| CDK4 | ns | | ns | ns | 1.76E-06 | ns | 6.98E-03 |
| CDCA3 | ns | | ns | ns | ns | ns | 2.96E-02 |
| HMGB2 | 1.76E-03 | | 3.67E-11 | ns | 1.52E-02 | ns | 1.29E-09 |
| RRM2 | 6.82E-08 | | 3.65E-27 | ns | 5.65E-13 | 8.82E-05 | 3.60E-22 |
| CKS2 | ns | | 5.25E-09 | ns | 1.39E-03 | ns | 5.51E-03 |
| E2F1 | ns | | 7.31E-08 | ns | 2.45E-28 | ns | ns |
| CDC6 | ns | | 5.9E-03 | ns | ns | ns | 1.23E-04 |
| CDC20 | ns | | 1.86E-08 | ns | 1.33E-02 | 2.57E-05 | 2.5E-04 |
| MCM7 | ns | | 3.21E-04 | ns | ns | ns | ns |
| MCM6 | ns | | 3.97E-02 | ns | 3.47E-03 | 3.17E-02 | 3.44E-03 |
| MAD2L1 | ns | | 3.09E-02 | ns | ns | ns | ns |
| CCNB2 | ns | | 6.22E-06 | ns | ns | 4.22E-02 | ns |
| CDKN2C | ns | | ns | ns | 2.24E-02 | ns | ns |
| CDK2 | ns | | 1.53E-02 | ns | ns | ns | ns |
